# Malignant peritoneal mesothelioma presenting as recurrent adhesion obstruction in general surgery: a case report

**DOI:** 10.1186/1752-1947-5-420

**Published:** 2011-08-30

**Authors:** Vijay Naraynsingh, Michael J Ramdass, Crystal Lee Lum

**Affiliations:** 1 Department of Surgery, General Hospital, Port of Spain, Charlotte Street, Port-of-Spain, Trinidad, West Indies

**Keywords:** peritoneal mesothelioma, clinical appearance

## Abstract

**Introduction:**

Malignant peritoneal mesothelioma is a well-described entity in many reports in the literature in which it has been associated with asbestosis. However, there is no information describing the gross appearance and cardinal features seen during laparotomy, hence it is easy for the unwary surgeon to miss the diagnosis of this rare condition.

**Case presentation:**

A 49-year-old man of African descent presented to our hospital with a three-month history of weight loss, anorexia, abdominal distension, and general signs of cachexia and ascites on second presentation. At first presentation one year prior to this, he had undergone a laparotomy at our institution by a different team for intestinal obstruction secondary to adhesions with no biopsy taken. The patient's condition subsequently progressively deteriorated, and investigations including upper and lower gastrointestinal endoscopies and computed tomography of the abdomen were inconclusive, except for some free fluid in the peritoneal cavity and diffuse, mild thickening of the gut wall and mesentery. A second-look exploratory laparotomy revealed widespread nodular thickening of the visceral peritoneum with a striking, uniformly diffuse, erythematous, and velvety appearance. The peritoneal biopsy histology showed that the patient had malignant peritoneal mesothelioma. His condition deteriorated rapidly, and he died eight weeks after surgery.

**Conclusion:**

Our report aims to increase the diagnosing clinician's awareness of the cardinal features of malignant peritoneal mesothelioma and thus reduce diagnostic errors and delays in treatment.

## Introduction

We present the case of a 49-year-old man with asbestos exposure to illustrate the rarity and difficulty of the diagnosis of malignant peritoneal mesothelioma (MPM). This case report focuses on the clinical appearance of the condition during exploratory laparotomy and demonstrates the striking, uniformly diffuse, erythematous, and velvety aspect of the tumor as it infiltrates the peritoneal surface. This presentation has not been described previously in the literature, and we hope that this information assists clinicians and surgeons in recognizing the condition, should they confront it in the future.

## Case presentation

At first presentation, a 49-year-old man of African descent who was a non-smoker presented with sudden onset of vomiting and abdominal distension. A clinical diagnosis of small-bowel obstruction was made based on presentation and plain abdominal radiographs. The patient underwent an exploratory laparotomy and a diagnosis of adhesions without obvious obstruction was diagnosed, but no biopsy taken.

**Figure 1:**
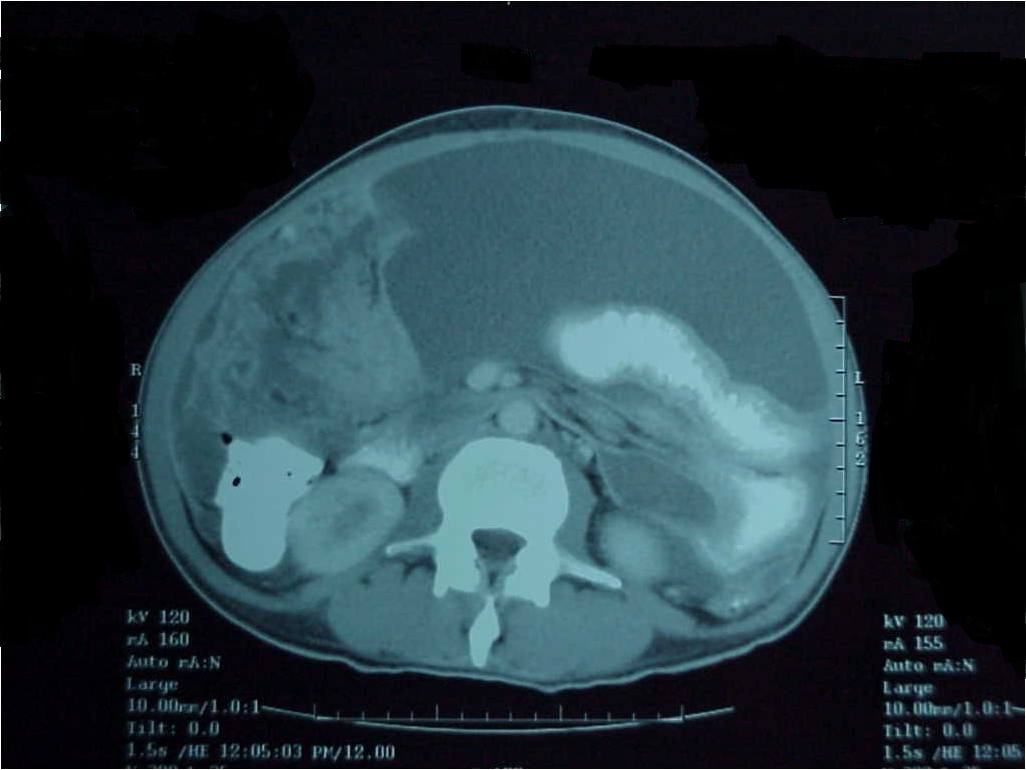
**Computed tomography scan with intravenous contrast enhancement showing the thickened gut wall and mesentery with the presence of ascites**.

He presented on a second occasion to our unit with progressive weight loss, anorexia, abdominal distension, and general signs of cachexia and ascites three months after his first laparotomy. We performed upper and lower gastrointestinal endoscopies as well as a computed tomography (CT) scan of the abdomen. These procedures produced no positive findings except for some free fluid in the peritoneal cavity and diffuse, mild thickening of the gut wall and mesentery. A detailed social history revealed that he had been exposed to asbestos between the ages of 23 and 29 years when he worked in a pipe-insulating factory. A CT scan obtained during his second presentation illustrated a thickened gut wall and mesentery with the presence of ascites (Figure 1), but all of his blood test results were normal.

**Figure 2:**
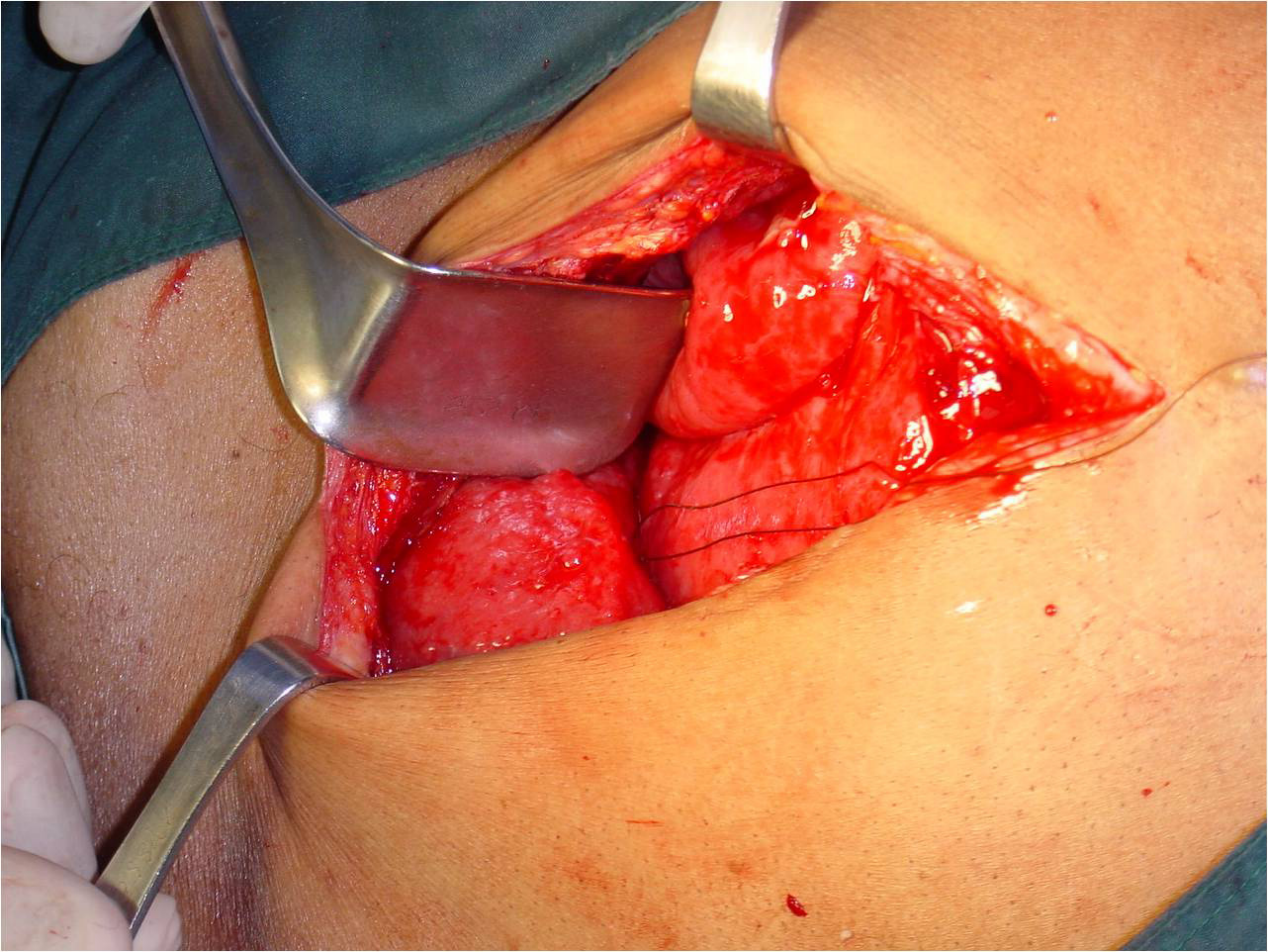
**Gross appearance of malignant peritoneal mesothelioma with widespread nodular thickening of the visceral peritoneum and a striking, uniformly diffuse, erythematous, and velvety appearance**.

**Figure 3:**
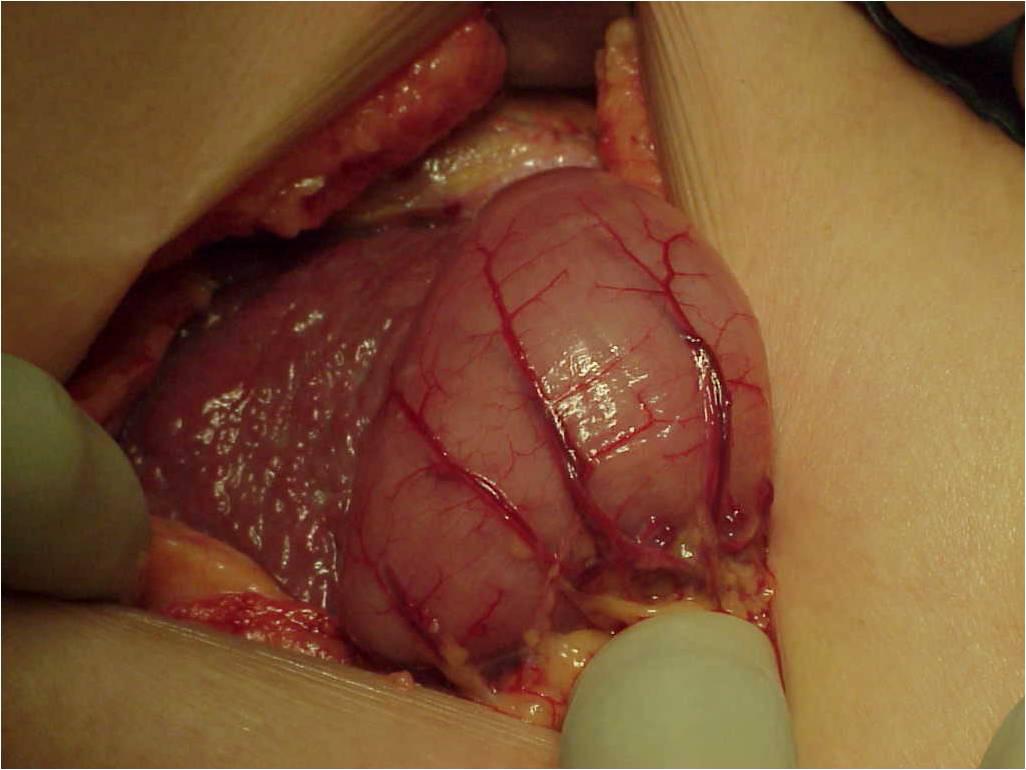
**Gross appearance of malignant peritoneal mesothelioma with widespread nodular thickening of the liver capsule**.

A second-look exploratory laparotomy revealed widespread nodular thickening of the visceral peritoneum with a striking diffusely erythematous and velvety look (Figures 2 and 3). His peritoneal biopsy histology showed this to be MPM. Cytoreduction was not done, since the disease was very diffuse and advanced at this time, with no focal mass, and hyperthermic intra-peritoneal chemotherapy was not available at our institution at the time. Adjuvant systemic chemotherapy was administered; however, the patient's condition worsened rapidly, and he died eight weeks after surgery.

## Discussion

MPM is a rare condition resulting most commonly from exposure to asbestos. The neoplasm manifests approximately 20 or more years after the initial exposure [1]. Pathologically, patients present with tumors of the pleura (pulmonary) and peritoneum and, less frequently, the pericardium and the tunica vaginalis [1]. Owing to the rarity and aggressive nature of this disease, the correct diagnosis is often delayed and treatment is frequently palliative.

MPM accounts for 20% to 33% of mesotheliomas, and histologically these may be divided into three basic forms: epithelioid (most common), sarcomatoid, and biphasic (mixed). Papillary peritoneal mesothelioma is a subdivision of the epithelioid form [2].

Clinically, this condition may be associated with abdominal pain, abdominal distension, ascites, weight loss, and pyrexia of unknown origin [3]. The latter is a rare presentation. Upon consideration of MPM in the differential diagnosis, the medical practitioner should proceed with the following investigations. A plain chest radiograph may show signs of asbestos in the lung: small opacities in the lower lung fields with or without pleural thickening or effusion, indicative of pleural mesothelioma [4]. An abdominal CT scan examination may show the presence of ascitic fluid and peritoneal thickening [5], and calretinin immunostaining of ascitic fluid can be done. This procedure has significantly increased the accuracy of the diagnosis [6].

At laparotomy, gross examination of the peritoneum may also reveal signs of the condition. Widespread nodular thickening of the visceral peritoneum with a striking, diffusely uniform, erythematous appearance (Figures 2 and 3) can be confirmed to be MPM on the basis of biopsy and histological examination. Grossly, peritoneal carcinomatosis can be confused with MPM. However, in carcinoma, the nodules vary considerably in size and the intervening areas are essentially normal (not uniformly erythematous). Peritoneal tuberculosis may also be confused with MPM, but in peritoneal tuberculosis the lesions are white with surrounding erythema and normal-appearing intervening serosa. In adhesions, the bands are obvious and the bowel serosa is normal.

In the case of our patient, the first surgeon unknowingly diagnosed adhesions without entertaining the diagnosis of MPM, probably because MPM is so rare that few surgeons would have suspected it. Therefore, no biopsies were taken during the first laparotomy.

Thus, if there is widespread nodular thickening of both parietal and visceral peritoneum with marked uniformly diffuse erythema and ascites, MPM should be suspected and a biopsy should be performed. Moreover, since exposure to asbestos often occurs many years before its effects manifest as overt disease, the patient does not usually reveal it while the history is being recorded unless specifically asked.

Once the diagnosis is confirmed by histology, multi-disciplinary management produces the best outcome [7]. The prognosis for patients with MPM is poor, with a survival time of approximately two years from diagnosis. Despite this poor prognosis, a potential cure has been described in the literature, the emphasis of which is early diagnosis coupled with definitive local and regional treatment.

Treatment options include operative cytoreduction, followed by heated intra-peritoneal chemotherapy applied intra-operatively with doxorubicin and cisplastin [8]. Early postoperative adjuvant therapy with paclitaxel is also initiated. The efficacy of these interventions are assessed during second-look cytoreduction surgery [9]. A recent paper by Husain *et al*. [10] highlights the fact that MPM is a rare tumor that is difficult to diagnose and provides guidelines for pathologists.

## Conclusion

This case report is important to public health, since it deals with the detection and diagnosis of MMP, which is a very important public health issue. It illustrates the vague clinical presentation of MPM, where the difficulty in diagnosis is a result of the rarity of this disease. Despite this fact, our patient presented with the most common symptoms of this condition. On the basis of the patient's history, occupational exposure to asbestos was a critical factor in the diagnosis. Furthermore, if the surgeon who performed the first laparotomy one year prior to the patient's second presentation had diagnosed MPM based on the appearance and examination of the peritoneum, this would have played a crucial role in early detection of the disease.

Consequently, we wish to highlight the clinical gross appearance of this rare condition for the unwary clinician as widespread fine nodular thickening of the visceral peritoneum with a striking, uniformly diffuse, erythematous, and velvety appearance. In contrast, other considerations in the differential diagnosis of this condition could be as follows: tuberculosis may present as ileocecal inflammation, carcinomatosis appears as hard white nodules with the intervening peritoneum having a normal appearance, and endometriosis of the peritoneum and/or omentum, characterized by hemorrhagic, reddish brown, or blue nodules or cysts on the peritoneal surface [10].

## Consent

Written informed consent for publication from the patient's next of kin could not be obtained despite all reasonable attempts. The case is important to public health and every effort has been made to protect the identity of our patient. There is no reason to believe that our patient would object to publication

## Competing interests

The authors declare that they have no competing interests.

## Authors' contributions

VN operated on the patient and was the major contributor to the idea of writing the article. MJR wrote and edited the manuscript. CLL assisted in researching the literature and writing the paper.
